# Supplementary figures and images for: Unveiling the Secretome of the Fungal Plant Pathogen Neofusicoccum parvum Induced by In Vitro Host Mimicry
Source: J Fungi (Basel). 2022 Sep 17;8(9):971. doi: 10.3390/jof8090971 (PMC9505667; doi:10.3390/jof8090971)

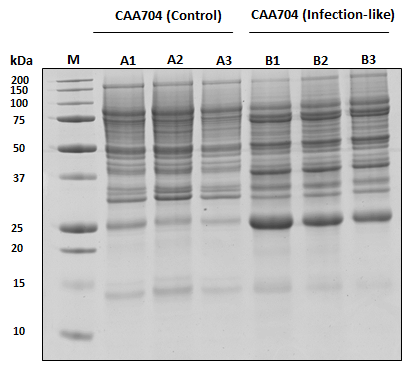

Supplement: Supplementary file 1 [file jof-08-00971-s001.zip › Figure S1.png]

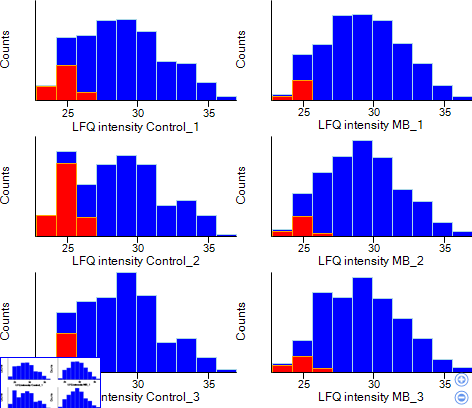

Supplement: Supplementary file 1 [file jof-08-00971-s001.zip › Figure S2.png]

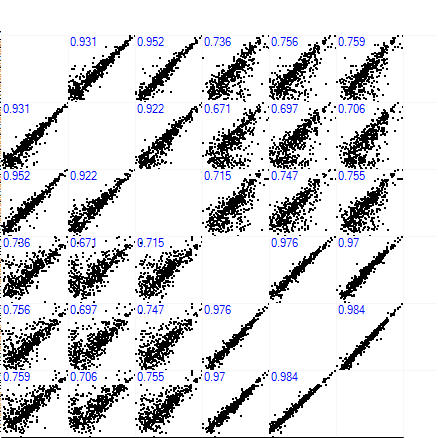

Supplement: Supplementary file 1 [file jof-08-00971-s001.zip › Figure S3.png]

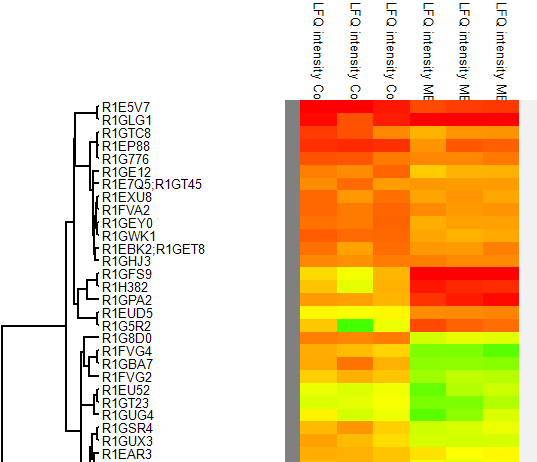

Supplement: Supplementary file 1 [file jof-08-00971-s001.zip › Figure S4.png]

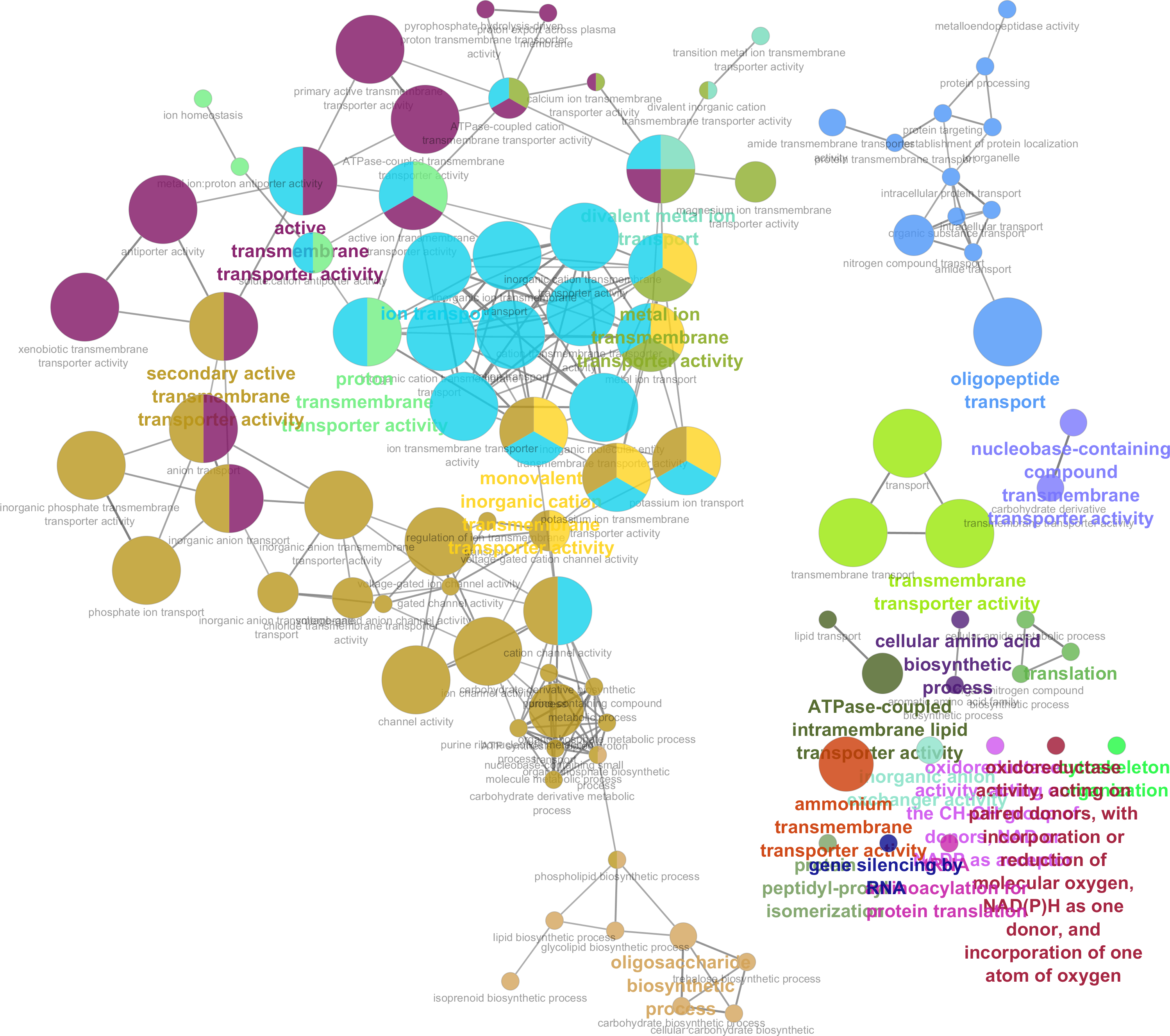

Supplement: Supplementary file 1 [file jof-08-00971-s001.zip › Figure S5A.png]

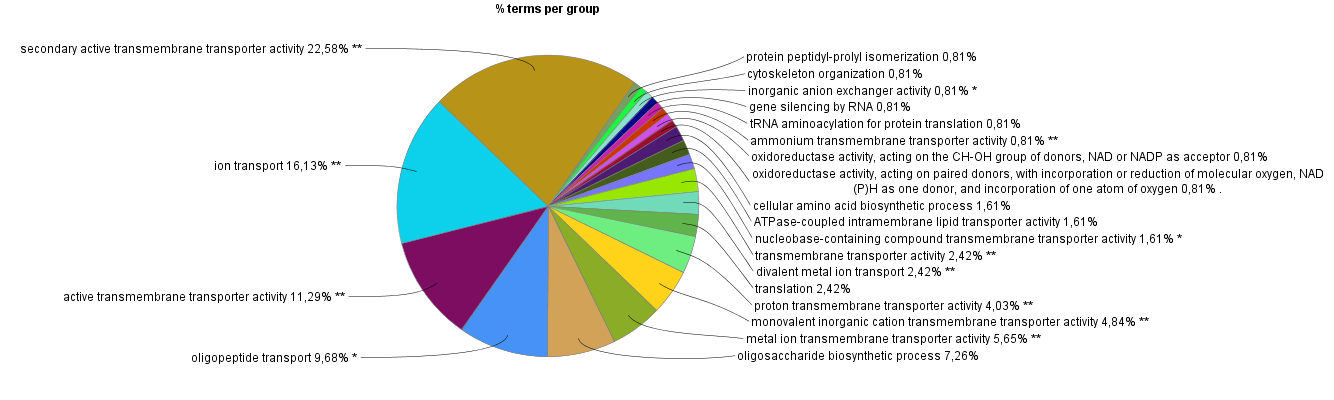

Supplement: Supplementary file 1 [file jof-08-00971-s001.zip › Figure S5B.png]

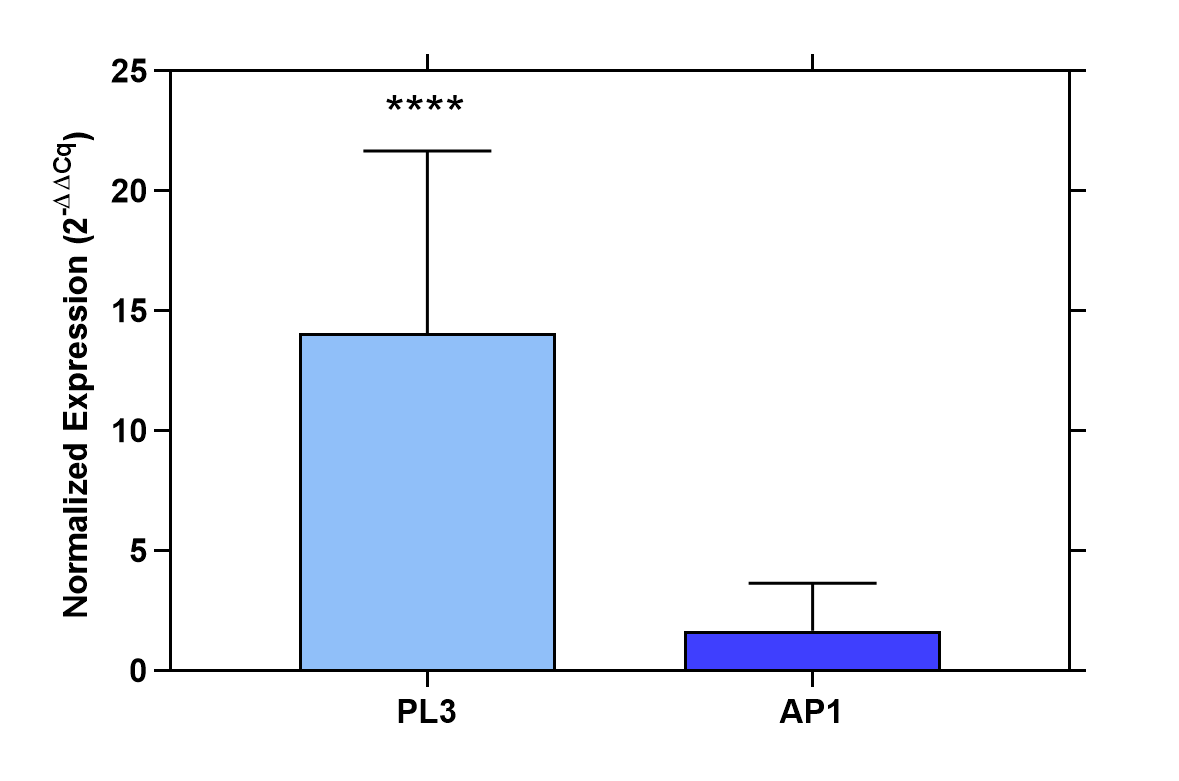

Supplement: Supplementary file 1 [file jof-08-00971-s001.zip › Figure S6.tif]
